# A vibration-based approach to quantifying the dynamic elastance of the superficial arterial wall

**DOI:** 10.1186/s12938-016-0147-4

**Published:** 2016-04-16

**Authors:** Jia-Jung Wang, Shing-Hong Liu, Hung-Mao Su, Steven Chang, Wei-Kung Tseng

**Affiliations:** Department of Biomedical Engineering, I-Shou University, Kaohsiung, 8, Yida Rd., Yanchao District, Kaohsiung, 82445 Taiwan, ROC; Department of Computer Science and Information Engineering, Chaoyang University of Technology, 168, Jifeng E. Rd., Wufeng District, Taichung, 41349 Taiwan, ROC; Department of Medical Research, China Medical University Hospital, China Medical University, 91, Xueshi Road, Taichung, 40402 Taiwan, ROC; Department of Cardiology, E-Da Hospital, 1, Yida Rd., Yanchao District, Kaohsiung, 82445 Taiwan, ROC

**Keywords:** Arterial stiffness, Dynamic elastance, Minute vibration method

## Abstract

**Background:**

The purpose of this study is to propose a novel method for assessing dynamic elastance of the superficial arterial wall using the sinusoidal minute vibration method.

**Methods:**

A sinusoidal signal was used to drive a vibrator which induced a displacement of 0.15 mm with a frequency range between 40 and 85 Hz. The vibrator closely contacted with the wall of a superficial radial artery, and caused the arterial wall to shift simultaneously. A force sensor attached to the tip of the vibrator was used to pick up the reactive force exerted by the radial arterial wall. According to the Voigt and Maxwell models, a linear relationship was found between the maximum reactive force and the squared angular frequency of the vibration. The intercept of the linear function represents the arterial wall elastance. In order to validate the feasibility of our method, twenty-nine healthy subjects were recruited and the wall elastances of their radial arteries were measured at room temperature (25 °C), after a 5-min cold stress (4 °C) and a 5-min hot stress (42 °C), respectively.

**Results:**

After the 5-min cold stimulation, the maximum radial wall elastance significantly increased from 0.441 ± 0.182 × 10^6^ dyne/cm to 0.611 ± 0.251 × 10^6^ dyne/cm (p = 0.001). In the 5-min hot stress, the maximum radial wall elastance significantly decreased to 0.363 ± 0.106 × 10^6^ dyne/cm (p = 0.013).

**Conclusions:**

The sinusoidal minute vibration method proposed can be employed to obtain the quantitative elastance of a superficial artery under different thermal conditions, and to help assess the severity of arterial stiffness in conduit arteries.

## Background

Arterial stiffness is frequently associated with the risk of cardiovascular diseases, cerebral vessel disease, diabetes mellitus and end-stage renal disease [1–3]. Some studies have approved that the higher arterial stiffness is a diffuse disease process of the conduit arteries that probably initiates in childhood and young adult life [4] and has a crucial effect on arterial remodeling in coronary systems [5]. As a result, to detect the alteration in arterial stiffness before the emergence of clinical vascular illness, it may serve as an early prognostic warning for cardiovascular disease.

Several physical indices have been explored to indicate the severity of arterial stiffness in previous investigations. Indices used for assessing global arterial stiffness include the pulse pressure [6], the capacitive compliance of large arteries and the oscillatory compliance of small arteries [7]. Systemic stiffness indices consist of the pulse wave velocity [8], characteristic impedance [9], carotid intima-media thickness [8], augmentation index of blood pressure [10] and β variables [11]. Furthermore, there were different indices to describe the local arterial stiffness, such as the arterial compliance [12], arterial distensibility [13], volume elastic modulus [14], Young’s elastic modulus [15], and spring constant of arterial wall [16].

It is more important to explore reliable techniques for the arterial stiffness measurement, in which the challenge is how to detect the absolute arterial diameter or the change of arterial diameter. Every technique usually has its own drawbacks and advantages. In the ultrasound echo-tracking technique [17], although vascular diameter (or blood flow velocity) can be measured, an individual training in the principles and technical skills of ultrasonography would affect its precision and accuracy. In the optical plethysmography [18], the change of vascular volume can be approximately computed since constituents of tissue have different absorption coefficients of light. The disadvantage is that it is difficult to calibrate the changed volume. Similarly, change in vascular volume can be measured by the impedance plethysmography based on different electrical characteristics of tissue [19, 20]. In the tonometry [21], the interaction between the peripheral artery and the water chamber of the tonometer, represented the change of arterial volume. In the oscillometry [12, 22], the cuff model was used to transfer the pulse pressure amplitude to the pulse volume amplitude.

Some studies used the indirect method to assess the arterial compliance. In the analysis of pulse transit time [23], according to the Moens–Korteweg relationship, the pulse wave velocity between two measured points may be proportional to the square root of arterial wall elastance, under the assumption that the wall thickness and lumen radius of the arteries are considered as constants. In the analysis of pressure waveform [24], the exponentially decayed phenomenon during the diastolic pressure waveform was found to be related to the arterial compliance.

Most of the techniques described above can only provide a rough arterial compliance index for either a part of an artery or for the whole artery system. Furthermore, the arterial characteristic apparently is not a constant, but dependent on the transmural pressure [12, 25]. Therefore, the purpose of the present research was to develop a novel non-invasive vibration-based method for determining the dynamic elastance of the radial artery. The arterial wall elastance was an absolute value and had the unit which could be used to describe the arterial stiffness condition.

## Methods

### Fundamental theory

**Fig. 1:**
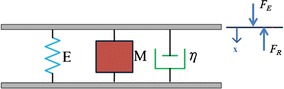
A three-component arterial model that characterizes the wall viscoelastic properties. E is an elastance component; M is an effect mass; η is a viscosity component; x is a displacement of the arterial wall; *F*_*E*_ is an external force; *F*_*R*_ is a reactive force

On the basis of Voigt and Maxwell models [26], a three-component mechanical model of an arterial segment was established and employed to characterize the viscoelastic properties of the arterial wall in the study. As shown in Fig. 1, the lumped arterial viscoelastic model consists of three parallel components which correspond to the mass (M) (or inertia), viscosity (η), and elastance (E) of the arterial wall, respectively. In this model, the elastance component was reasonably assumed to be a function of time or transmural pressure. When an external sinusoidal vibration force (*F*_*E*_), perpendicular to the axis of an arterial lumen, contacted with the outside of the arterial wall and created the arterial wall displacement, *x*, the arterial wall would generate a reactive force (*F*_*R*_) in response to the external force. According to the force balance, the reactive force should be equal to the summation of the inertia-related force, viscosity-related force and elastance-related force [27], as follows:

1 $$F_{R} (t) = \frac{{d^{2} x(t)}}{d(t)} + \eta \frac{dx(t)}{dt} + E(t)x(t).$$

When the external force was of the sinusoidal vibration, the displacement of the arterial wall also was a sinusoidal function. It can be written as:

2 $$x(t) = A_{m} \sin (\varpi t),$$

where *A*_*m*_ is the peak amplitude and *ω* is the angular frequency. Furthermore, its first and second derivatives can be respectively expressed as:

3 $$\frac{dx(t)}{dt} = A_{m} \varpi \cos (\varpi t),$$

and

4 $$\frac{{d^{2} x(t)}}{dt} = - A_{m} \varpi^{2} \sin (\varpi t).$$

According to Eqs. (2), (3) and (4), Eq. (1) can be changed to

5 $$\frac{{F_{R} (t)}}{{A_{m} }} = [E(t) - M\varpi^{2} ]\sin (\varpi t) + \eta \varpi \cos (\varpi t).$$

In order to simplify Eq. (5), we know that

$$\begin{aligned} x(t) &= A_{m} \sin (\omega t) = A_{m} ,\quad \omega t = \pm \frac{(n + 1)\pi }{2},\quad n \in i, \\ \frac{dx(t)}{dt} &= A_{m} \cos (\omega t) = 0, \quad \omega t = \pm \frac{(n + 1)\pi }{2},\quad n \in i, \end{aligned}$$

where *i* is an integer. Then, we define *T*_*m*_ as: $T_{m} = \frac{\pi }{2\omega }.$ When *t* is *T*_*m*_, Eq. (5) can be simplified and *F*_*R*_ is equal to the maximum value, *F*_*R_max*_,

6 $$\frac{{F_{R} (T_{m} )}}{{A_{m} }} = \frac{{F_{R\_\text{max} } }}{{A_{m} }} = E(T_{m} ) - M\omega^{2}$$

According to Eq. (6), at the specific time *T*_*m*_ the ratio of *F*_*R_max*_ and *A*_*m*_ is negatively and linearly proportional to the square of angular frequency (*ω*^2^). In this linear polynomial function, *E*(*T*_*m*_) is the intercept and *M* is the absolute slope.

### Measuring system

**Fig. 2:**
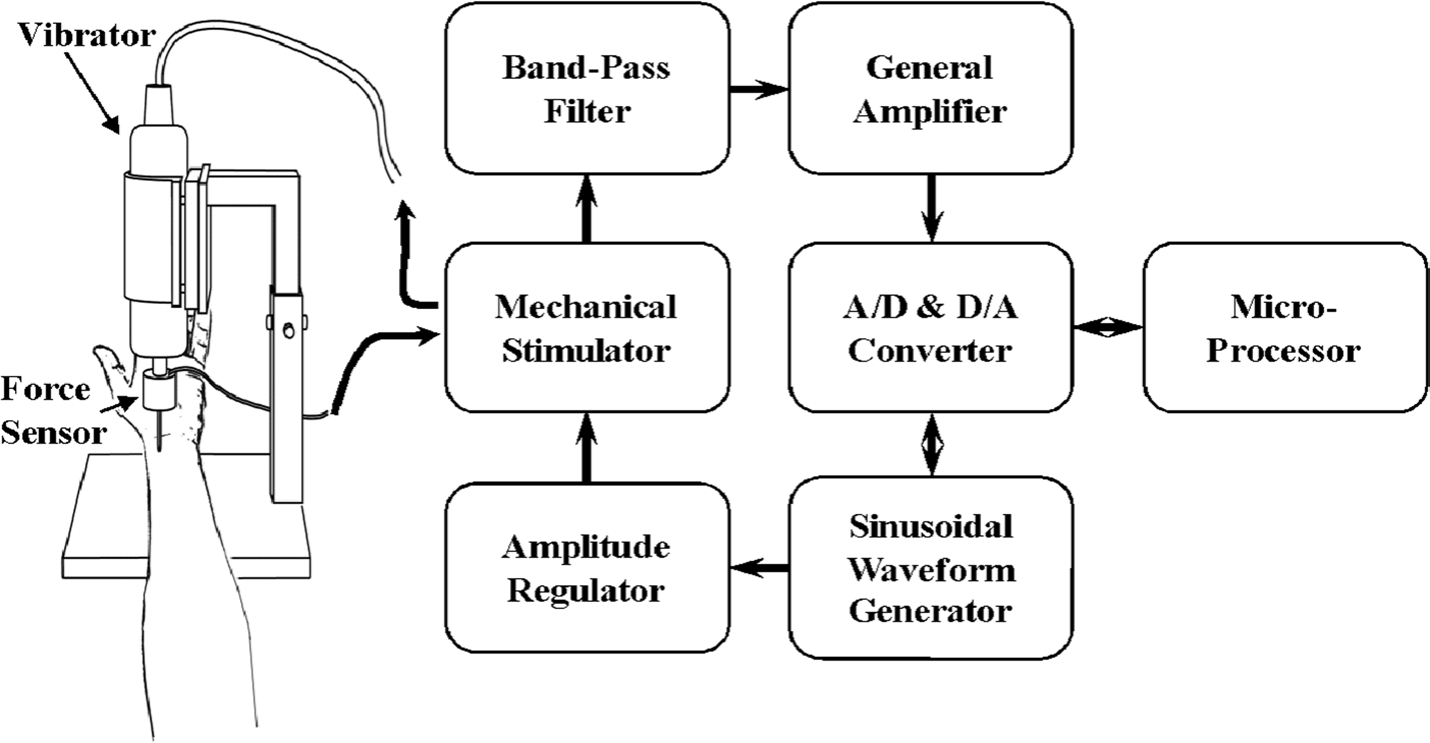
Schematic diagram of the dynamic elastance measurement system

Figure 2 shows the schematic diagram of the designed measuring system in this study. The system consists mainly of a vibrator, a force sensor, a vibrator driver, a sinusoidal waveform generator, a sensor driver, and an MP100 system. A sinusoidal signal delivered by the sinusoidal waveform generator was used to trigger the vibrator driver (DPS-270, DiaMidical System Cor., Tokyo, Japan). The vibrator driver drove the vibrator and the reactive force was sensed by a force sensor placed at the tip of the vibrator. Then, the reactive force signal was converted to a digital signal by the MP100 system (BIOPAC System, Inc., USA). The resolution is 12 bits and the sampling rate is 1000 Hz. For practical applications, the peak displacement of the sinusoidal vibration was set to 0.15 mm and the contact area of the vibrator with the radial arterial surface was about 0.2 cm^2^. The data processing was performed with the AcqKnowledge Software 3.9 (BIOPAC System, Inc., USA).

### Experimental protocol

Twenty-nine healthy college volunteers (17 M and 12 F, age: 23 ± 3 years, systolic pressure: 113 ± 14 mm Hg, diastolic pressure: 70 ± 10 mm Hg, heart rate: 73 ± 9 beats/min) participated in this study. The clinical trial was approved by the Institutional Review Board of the E-DA Hospital, Kaohsiung, Taiwan (no. EMRP61101 N), and an informed consent was obtained from each participant prior to the initiation of the study. The temperature in the research lab was maintained at 25 °C with air-conditioners. Each subject was put in a sitting position and was asked to take a 5-min rest before the measurement. We measured the blood pressure before and after cold and hot stress tests by OMRON blood pressure monitor. During the measurement, each subject was asked to sit on an adjustable-height chair. The subject’s left hand was placed on a table at the same horizontal height as the heart with the palm pointing upwards and the wrist resting on a soft pillow. The superficial part of the radial artery of the left hand was forced up by the vibrator with a peak displacement of 0.15 mm. The frequency of the vibration was increased from 40 to 85 Hz at a step of 5 Hz. In the experiment, there were three phases, a baseline test, a cold stress test, and a hot stress test. In the baseline test, subjects were measured at room temperature. In the cold stress test, a plastic bag containing a mixture of ice and water (around 4 °C) was put on the inside surface part of the left forearm. Subjects were measured after the 5-min cold stress. Then, subjects were asked to take a 5-min rest in order to make the subjects’ hemodynamic variables stable. Another plastic bag filled with hot water at 42 °C was placed on the left forearm of the subject lasting for 5 min, and subjects were measured again.

### Data processing

**Fig. 3:**
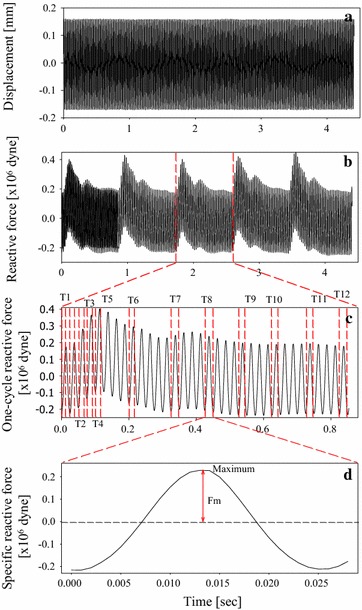
Explanation for acquiring maximum reactive force (*Fm*). **a** Sinusoidal displacement of 40 Hz; **b** Reactive force of the arterial wall in response to the sinusoidal vibration; **c** Reactive force signal of one-cardiac cycle extracted from (**b**); **d** One-vibration cycle reactive force waveform extracted form (**c**)

For different sinusoidal frequencies, 12 specific time sections (indicated by T1–T12) within one cardiac cycle were selected based on the ECG and the reactive force signals. Each time section was a vibration cycle. The fifth time section (T5) was set at the maximum amplitude cycle of the reaction force signal within the systolic duration, and T1 and T12 were set at the beginning and end cycles of the reactive force signal, respectively. The duration between T1 and T5 and between T5 and T12 were both equally divided. T2, T3, and T4 were selected within T1–T5, and T6, T7, T8, T9, T10, and T11 were chosen within T5–T12. Then, the peak amplitude of the reactive force signal in each time section was defined as the *F*_*R_max*_. As explanation, Fig. 3 demonstrates how to determine the *F*_*R_max*_ of the reactive force at the specific time section. A sinusoidal vibration with 40 Hz forced the radial artery, as shown in Fig. 3a. Meanwhile, a reactive force signal from the arterial wall was measured and displayed in Fig. 3b. The reactive force signal within one cardiac cycle was shown in Fig. 3c. Then, the one-cycle reactive force within the T8 was displayed in Fig. 3d. The peak amplitude of the reactive force was found within this time section. Therefore, each time section had one *F*_*R_max*_. Since it was difficult to control human body movement in the measuring duration, five values of *F*_*R_max*_ extracted from five-cycle reactive force signal were averaged to yield one typical *F*_*R_max*_. Similarly, other wall elastances corresponding to different time sections were determined using their average. We did this measurement again after the frequency of the vibrator was changed.

**Fig. 4:**
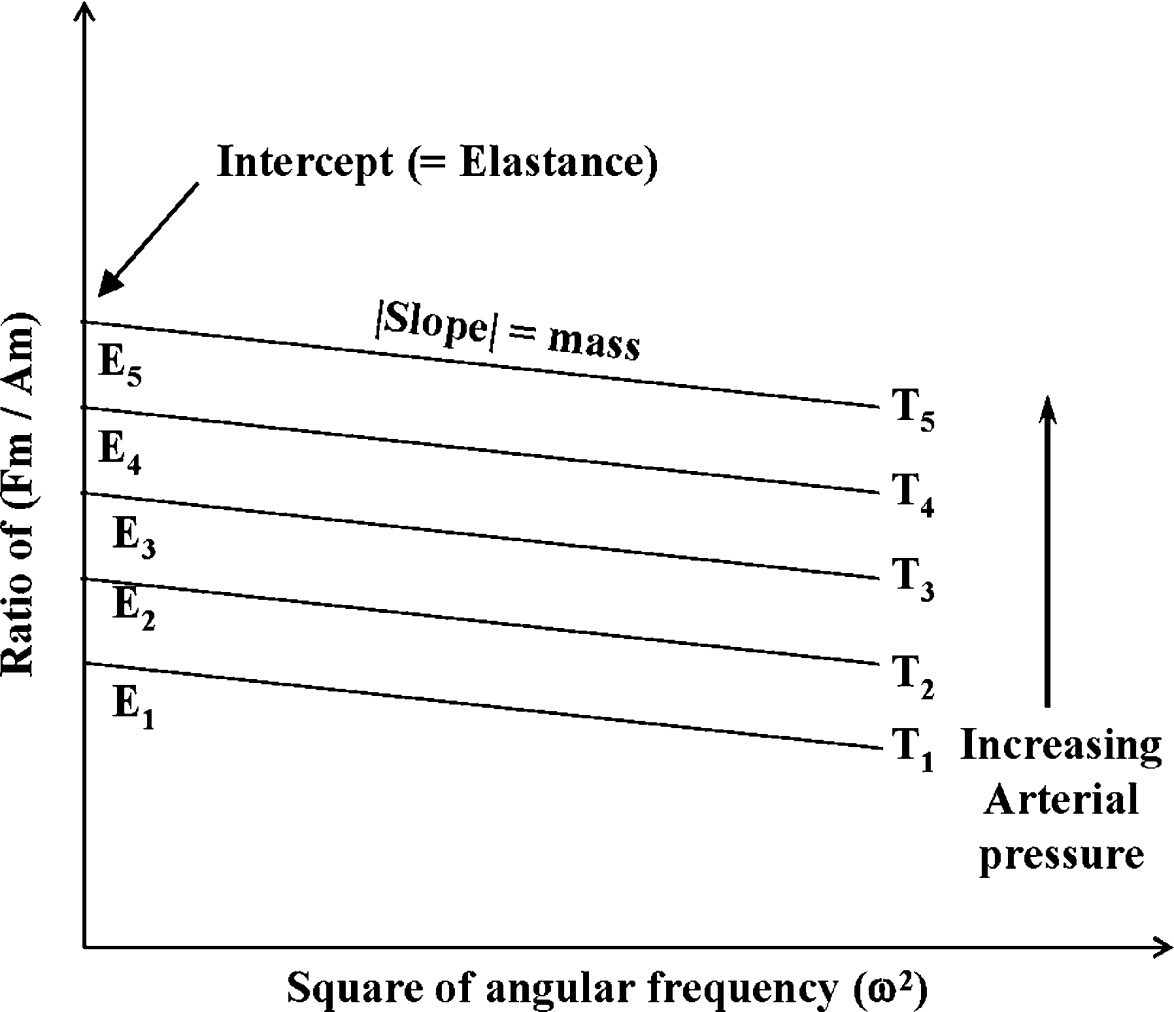
The elastances of the arterial wall were estimated by Eq. (6). The intercept represents *E* and the slope represents *M*.F_R_max_ = the maximum reactive force; Am = the maximum amplitude of sinusoidal displacement

According to Eq. (6), *F*_*R_max*_ and ω^2^ have a linear relation which is illustrated in Fig. 4. Because we only considered the systolic duration, the five specific time sections (T1, T2, … and T5) were used to estimate the different elastances of the arterial wall (E1, E2,… and E5) under the different transmural pressure. It is worthwhile noting that all lines in Fig. 4 are in parallel and have the same slope that equals to the effective mass.

### Statistical analysis

The quantitative data are expressed as mean ± SD. To compare the changes of the elastance in the cold stress and hot stress with those at room temperature, a 2-tailed paired *t* test was used. A p value of 0.05 or less was considered statistically significant. Also, the degree of linear relationship between the two variables was represented by correlation coefficient in linear regression analysis using Sigma Plot 11.0 (Systat Software, Inc., USA).

## Results

**Fig. 5:**
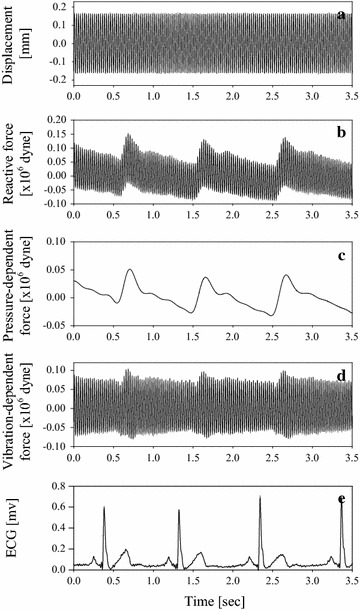
Typical time course of (**a**) the vibrator signal with 0.15 mm displacement and 40 Hz vibration, **b** the reactive force signal, **c** the arterial pressure-dependent force without the vibrator effect, **d** the sinusoidal vibration-dependent force, **e** the electrocardiogram (ECG) signal

Table 1 shows the blood pressure before and after the cold and hot stress tests. We found in general that both the systolic and diastolic blood pressures were significantly increased during the cold stress, but decreased during the hot stress in the 29 volunteers. A vibrator with 0.15 mm displacement and 40 Hz vibration was used to force the wall of the radial artery as shown in Fig. 5a, and a typical reactive force signal detected by the force sensor is illustrated in Fig. 5b. Theoretically, the reactive force included two components. One is called the pressure-dependent force that can be extracted from the reactive force signal by a four order lowpass filter with a cutoff frequency of 20 Hz. This pressure-dependent force is generated by the arterial blood pressure expanding the arterial wall, as displayed in Fig. 5c. The other is called the vibration-dependent force, which is induced by the vibrator, as shown in Fig. 5d. This vibration-dependent force can be obtained from the reactive force signal subtracting the pressure-dependent force signal. In order to extract the systolic duration, the ECG signal in Fig. 5e is used as a time reference.

**Table 1:** Comparison of arterial blood pressure measured before and after thecold and hot stresstests

Parameters	Cold stress (4 °C)		Hot stress (42 °C)	
	Before	After	Before	After
Systolic pressure (mmHg)	113 ± 14	117 ± 16* (p = 0.021)	123 ± 16	118 ± 17** (p = 0.005)
Diastolic pressure (mmHg)	70 ± 10	74 ± 10* (p = 0.016)	74 ± 12	71 ± 12** (p = 0.008)
Heart rate (beats/min)	73 ± 9	72 ± 9 (p = 0.647)	75 ± 12	71 ± 9** (p = 0.003)

* p < 0.05, ** p < 0.01

**Fig. 6:**
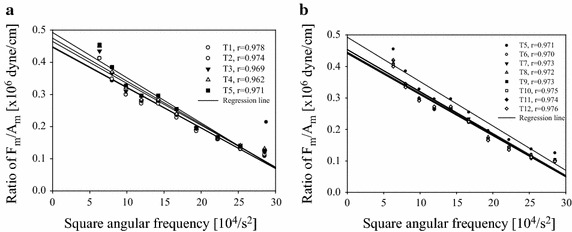
The linear relationship between ω^2^ and F_R_max_/Am in the systolic and diastolic durations of one heart beat was used to determine the arterial wall elastance. The data was taken from Fig. 5. **a**The five regression lines correspond to T1–T5 in the systolic duration, respectively. **b** The eight regression lines correspond to T5 and to T12 in diastolic duration, respectively.The intercept of the linear regression represents the arterial wall elastance. Different *dot symbols* denote the experimental data and the *solid lines* are the regression lines with this data

Figure 6a demonstrates the linear relationship between ω^2^ and F_R_max_/Am in the systolic duration of one heart beat at room temperature. The data was taken from Fig. 5. The best and worst linear correlation coefficients were 0.978 and 0.962 at T1 and T4, respectively. Also, it is worthwhile noting that the regression line at T5 had the largest intercept. It means that the largest arterial wall elastance occurred at T5 which was also the systolic blood pressure. On the contrary, the smallest arterial wall elastance happened at T1 which was the diastolic blood pressure. Similarly, Fig. 6b shows the linear relationship between ω^2^ and F_R_max_/Am in the diastolic duration of the same heart beat in Fig. 6a. The best and worst linear correlation coefficients were 0.975 and 0.970 at T11 and T7, respectively. It is clear that the largest and smallest intercepts happened at T6 and T12.

**Fig. 7:**
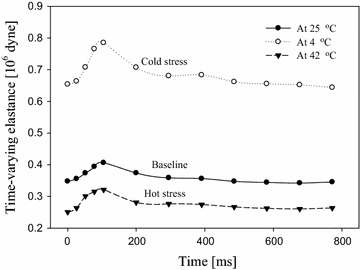
Typical elastance curves of one cardiac cycle measured from one subject at the cold stress, the baseline and the hot stress. *Data points* represent the measuring values of wall elastance

Figure 7 Shows three elastance curves determined at room temperature, after the 5-min cold stress and after the 5-min hot stress, respectively. Individual elastance curve was reconstructed using the 12 elastance values. In general, the elastance values with respect to the first five time sections (T1–T5) were gradually increased following the change of blood pressure. Also, the elastance values corresponding to the later seven time sections (T6–T12) were gradually decreased following the change of blood pressure. Furthermore, the largest elastance always happened at T5. It is worthwhile noting that the 5-min cold stimulation made the elastance curve to an upward shift. On the contrary, the elastance curve shifted down after the 5-min hot stimulation. However, these three elastance curves have similar changing tendency.

Table 2 summarizes the comparison of the arterial wall elastance obtained from the 29 subjects at three different thermal conditions. We used the arterial wall elastance at room temperature as the baseline of the experiment. The maximum and minimum elastance values were taken from T5 and T1. In the cold water stimulation, the elastance values were larger than the other experiments. Thus, the maximum and minimum elastance values (0.611 ± 0.251·10^6^, 0.520 ± 0.242·10^6^ dyne/cm) all had significant differences with the baseline elastance values (0.441 ± 0.182·10^6^, 0.378 ± 0.179·10^6^ dyne/cm). Their p values all were 0.001. The elastance values in the hot water stimulation were smaller than the other experiments. Therefore, the maximum and minimum elastance values (0.363 ± 0.106·10^6^, 0.299 ± 0.107·10^6^ dyne/cm) also had significant differences with the baseline elastance values. Their p values were 0.013 and 0.014, respectively.

**Table 2:** Comparison of arterial wall elastance determined in cold stress, room temperature and hot stress conditions

Parameters	Room temp (25 °C)	Cold stress (4 °C)	Hot stress (42 °C)
Maximum elastance (106 dyne/cm)	0.44 ± 0.182	0.611 ± 0.251*** (p = 0.001)	0.363 ± 0.106* (p = 0.013)
Minimum elastance (106 dyne/cm)	0.378 ± 0.179	0.520 ± 0.242*** (p = 0.001)	0.299 ± 0.107* (p = 0.014)
Change of elastance (106 dyne/cm)	0.063 ± 0.025	0.091 ± 0.038** (p = 0.002)	0.064 ± 0.028 (p = 0.812)

* p < 0.05; ** p < 0.01; *** p < 0.001

Both maximum elastance and minimum elastance were found to be significantly augmented by the 5-min cold stress (p = 0.001 for both), as compared with those at room temperature. Additionally, the short-term cold stress made the changed range of the arterial wall elastance considerably wider (p = 0.002). In contrast, the 5-min hot stress let the arterial wall elastance significantly decrease (p = 0.013 for maximum elastance, p = 0.014 for minimum elastance). But, the changed range of the arterial wall elastance was the same as the baseline.

## Discussion

In several previous investigations [12, 25] the arterial dynamic compliance follows the change of the transmural pressure. In our method, the reactive force included two forces, the pressure-dependent force and the vibration-dependent force. Although the two forces are time-variant amounts, we only detect the reaction force in each specific time sections. Thus, the vibration-dependent force was considered as a constant force. The change of transmural pressure followed the change of blood pressure. Therefore, in the study, we separated twelve time sections in one cardiac cycle. In Fig. 7, we found that the changes of the radial wall elastance follows the changes of blood pressure. The maximum and minimum elastance values happened at the systolic and diastolic pressures, separately. The results conformed to the Voigt and Maxwell models [26].

Wei et al. had used the theorem of spring constant to estimate the arterial elastance by the radial blood pressure waveform [16] and photoplethysmogram waveform [27]. In their study, they have proposed two hypotheses, the blood pressure forced the extension of the arterial wall and their relation was linear. Therefore, the displacement, velocity, and acceleration of the arterial wall movement were obtained from the blood pressure waveform. Then the spring constant of the arterial wall could be detected. But, this method has two problems. One is that the arterial wall elastance is not a constant. Using the change of blood pressure waveform to replace the displacement of the arterial wall is a rough transformation. Second, the spring constant in their method had no proper units. It only was called an “index of arterial stiffness”. But, in the present study, we used the ratio of *F*_*m*_ and *A*_*m*_ (dyne/cm) at the different trasmural pressures to describe the elastic characteristic of arterial wall as the arterial stiffness index.

Many non-invasive and non-image methods have been used to indirectly or directly estimate the local arterial stiffness or the systematic compliance, including pulse wave velocity [21], pulse wave analysis [10, 24], impedance plethysmography [11, 14], photoplethysmography [16], and oscillometry [12]. But, in these methods, the largest challenge is how to calibrate the parameter’s unit and measure the parameter’s absolute value. In our method, the force sensor and the vibrator all have been calibrated by the manufacturers. Thus, it is believed that the measured force and the moving distance of vibrator are reasonably accurate, and then the elastance (*F*_*m*_/*A*_*m*_) should be reliable. The measured elastance has a proper unit and is an absolute value which depends on the transmural pressure. However, the measured ratio of *F*_*m*_ and *A*_*m*_ could not fully describe the elastance of arterial wall because there are some mediums between the radial arterial wall and the tip of the sensing probe. The mediums really have some contribution (probably a DC component) to the measured elastance. Thus, the measured wall elastance by the current method can be considered as an arterial stiffness index which may be applied to evaluate the arterial stiffness condition. Other drawbacks of the present method include the difficulties in setting the optimal measurement location and in installing the measurement system, as well as the lack of a precise calibration procedure.

In order to validate the feasibility of our method, we did the 5 min cold and hot water stimulation trials to compare with the results at room temperature. In Table 1, it is found that the radial wall elastance significantly rose after the cold water stimulation, the mean of minimum radial wall elastance (0.520 ± 0.242·10^6^ dyne/cm) in the cold stress was greater than maximum radial wall elastance (0.441 ± 0.182·10^6^ dyne/cm) in room temperature. Moreover, it also significantly descended after the hot water stimulation, the mean of maximum radial wall elastance (0.363 ± 0.106·10^6^ dyne/cm) in the hot stress was smaller than the minimum radial wall elastance (0.378 ± 0.179·10^6^ dyne/cm) in room temperature. The response of the arterial wall elastance in these trials completely matched the physiological response.

The mediums between the radial arterial wall and the tip of the sensing probe have the skin, tissue, and muscle which could affect the accuracy of the arterial wall elastance measurement in this study. Thus, the tip of the vibrator and force sensor must be placed at the superficial radial artery. The area used on the wrist to measure the radial wall elastance is very small. Moreover, the displacement of the vibrator is very small (0.15 mm). If the subject’s hand has a slight motion, the reaction force has a big reaction. Thus, in the experiment, we continuously recorded many heart beat cycles, and extracted the most perfect signal to detect the wall elastance.

With the proposed method, it is clear that the higher the frequency of sinusoidal vibration, the better resolution to describe the dynamic elastic characteristics of arterial wall. However, it is very hard for a mechanical device to produce high-frequency minute vibrations without any distortion. In this study, when the vibration frequency became greater than 100 Hz, more distortion existed in the sinusoidal displacement waveform. Such distorted sinusoidal waveform will no longer satisfy the requirement of Eqs. (2)–(6). Therefore, we have to make a compromise between the selection of higher frequency and the distortion of the sinusoidal waveform in applying the proposed vibration method. After several trial and error experiments, we have done our best to adjust the frequency and waveform of the vibrator to yield the better results.

## Conclusion

The proposed vibration method is successfully applied to calculate the dynamic and absolute values of radial arterial wall elastance under different thermal situations. The measured wall elastance may be considered as a local arterial stiffness index and the elastance curve corresponding to an entire cardiac cycle, obtained with the novel method, may be helpful for diagnosis of peripheral arterial disease.
